# Diagnosis and management of Parkinson's disease dementia

**DOI:** 10.1111/j.1742-1241.2008.01869.x

**Published:** 2008-10

**Authors:** W Poewe, S Gauthier, D Aarsland, J B Leverenz, P Barone, D Weintraub, E Tolosa, B Dubois

**Affiliations:** 1 Department of Neurology, Medical University InnsbruckInnsbruck, Austria; 2 Alzheimer's Disease Research Unit, McGill Centre for Studies in Aging, Douglas Mental Health University InstituteMontréal, QC, Canada; 3 Norweigen Centre for Movement Disorders, Stavenger University HospitalStavenger, Norway; 4 Institute of Clinical Medicine, University of BergenBergen, Norway; 5 Mental Illness and Parkinson's Disease Research Education and Clinical Centers, VA-PSHCS, and Departments of Neurology and Psychiatry and Behavioral Sciences, University of WashingtonSeattle, WA, USA; 6 Dipartimento di Scienze Neurologiche, Università Federico II di NapoliNaples, Italy; 7 Department of Psychiatry, University of PennsylvaniaPhiladelphia, PA, USA; 8 Parkinson's Disease and Movement Disorders Unit, Institut Clínic de Neurociències, and Centro de Investigación Biomédica en Red sobre Enfermedades Neurodegenerativas (CIBERNED), Hospital Clínic de Barcelona, Institut d'Investigacions Biomèdiques August Pi i Sunyer, Universitat de BarcelonaBarcelona, Spain; 9 INSERM-UPMC UMRS 610, Federation of Neurology, Salpêtrière Hospital; University of ParisParis, France

## Abstract

Parkinson's disease (PD) has long been considered predominantly a motor disorder. However, its frequent association with dementia, which contributes significantly to the morbidity and mortality of the condition, is gaining increasing recognition. PD dementia (PDD) has a unique clinical profile and neuropathology, distinct from Alzheimer's disease (AD). Cholinergic deficits, a feature of both AD and PDD, underlie the rationale for cholinesterase inhibitor therapy in both conditions. In clinical practice, it is important that PDD should be recognised and appropriately treated. This review aims to outline the recently proposed clinical diagnostic criteria for PDD and to summarise the guidelines/recommendations published since 2006 on the use of cholinesterase inhibitors in the management of PDD. Although the cholinesterase inhibitor rivastigmine has recently been approved for the management of PDD, there remains a need for the development of novel therapies that can affect key mechanisms of the disease or prevent/delay patients with PD and mild cognitive impairment from progressing to PDD.

**Review Criteria**

This review focuses primarily on the clinical diagnostic criteria for PDD recently published by a Task Force of the Movement Disorder Society (MDS). In addition, guidelines/recommendations published since 2006 on the use of cholinesterase inhibitors for the management of PDD are summarised. Articles were identified using MEDLINE in January 2008 (search limits: last 5 years) using the terms: dementia; treatment; guidelines; and recommendations.

**Message for the Clinic**

A simple algorithm has been proposed to help clinicians to recognise and accurately diagnose PDD as a distinct dementia syndrome. Patients with this condition can benefit from treatment with cholinesterase inhibitors.

## Introduction

Parkinson's disease (PD) is a progressive neurodegenerative disease that affects 1–2% of people older than 60 years of age (1). Although PD has long been considered predominantly a motor disorder, its frequent association with dementia has recently gained increasing recognition (2–4). Patients with PD have an almost sixfold increased risk of developing dementia compared with age-matched individuals without PD (5). In a 12-year population study of patients with PD, the cumulative incidence of dementia increased steadily with age and disease duration reaching 80–90% by age 90 years (conditional on survival) (6). Dementia contributes significantly to the morbidity and mortality of PD (7,8). Key risk factors or correlates consistently associated with PD dementia (PDD) are older age, more severe parkinsonism (particularly rigidity, postural instability and gait disturbance), male gender, certain psychiatric symptoms (depression, psychosis) and mild cognitive impairment (MCI) (9–11).

Mild cognitive impairment is a condition that can occur as a transitional state between normal ageing and dementia and has traditionally been used to describe patients who frequently go on to develop Alzheimer's disease (AD) (12). An analogous concept of PD-MCI has been proposed and recent cross-sectional studies suggest that more than 20% of PD patients meet criteria for PD-MCI with a majority going on to develop PDD over time (13,14). Defining PD-MCI offers an opportunity for further study of cognitive impairment in PD and targets earlier therapeutic intervention.

The cognitive profile of PDD may be different from that of AD. Specifically, impairments in attention, executive and visuo-spatial functions tend to dominate in PDD, with memory encoding and language abnormalities playing a less significant role than they do in AD (2,3). A recent analysis comparing the profiles of cognitive impairment in 976 patients with AD or PDD suggested that diagnosis could be predicted from the cognitive profile with 74.7% accuracy (15). Worse performance by AD patients on the orientation task and PDD patients on the attentional task best distinguished the two diagnostic groups (15). Both groups showed memory impairment, although AD patients performed worse than PDD patients in this domain (15). Neuropsychiatric symptoms, common in both diseases, also present themselves characteristically, with visual hallucinations and rapid eye movement sleep behaviour disorders occurring much more frequently in PDD than in AD (16,17).

The classical motor features of PD include rigidity, resting tremor, bradykinesia and postural instability. These motor symptoms are believed to result from a gradual loss of dopaminergic neurons projecting from the substantia nigra to the striatum because of the deposition of Lewy bodies constituted of α-synuclein protein (18). However, the neuropathophysiological underpinnings of dementia in PD are a subject of continued debate (19). While AD pathology may contribute to PDD in some cases (20), recent research suggests that the neural substrate of most cases of PDD is Lewy body/synuclein pathology (21,22). Therefore, PDD appears to be distinct in terms of its clinical profile and neuropathology (19).

Nevertheless, both PDD and AD are associated with marked cholinergic deficits (to a greater extent in PDD than in AD) (23,24) and it is these deficits that underlie the rationale for cholinesterase inhibitor therapy in both conditions. The first clinical evaluation of a cholinesterase inhibitor in PDD comprised a small, open-label study of tacrine (25). The suggestion of clinical effectiveness in that study gave rise to a series of open-label trials and case series to assess donepezil (26–28), rivastigmine (29–31) and galantamine (32) in PDD. Two small double-blind, placebo-controlled trials appeared to demonstrate modest cognitive benefits for donepezil (33,34). However, only one large (*n*=541), double-blind, placebo-controlled cholinesterase inhibitor trial has been published to date (11). Statistically significant effects of rivastigmine capsules vs. placebo on a range of primary and secondary outcome measures were observed including cognitive performance, attention, executive function, activities of daily living (ADLs) and behavioural symptoms (11). In secondary analyses, these effects were particularly marked in patients with clinical markers predictive of a more aggressive course of disease, such as hallucinations (35) and elevated plasma homocysteine levels at baseline (36). Currently, donepezil, rivastigmine and galantamine are widely approved for the treatment of AD; rivastigmine is the only pharmacological agent currently approved for the treatment of PDD in Europe, the USA and Canada. Last year in the USA, a patch containing rivastigmine became the first transdermal treatment approved for both AD and PDD. In contrast to AD, trial data of memantine are not available for PDD.

In clinical practice, PDD often goes unrecognised and, as a result, is not appropriately treated. The expanding population of patients with PD (37), the recognition that dementia is a very common non-motor complication of PD and the recent FDA approval of a cholinesterase inhibitor (rivastigmine) to treat PDD have created a surge of interest in recognising, diagnosing and treating PDD. As a result, the Movement Disorder Society (MDS) recruited a task force comprising 23 members representing various disciplines and geographical regions, to propose clinical diagnostic criteria for PDD (10). In addition, several guidelines/recommendations on the use of different agents in the management of this condition have been published since 2006 (38–41). These guidelines and current evidence for the use of cholinesterase inhibitors in PDD are reviewed here.

## Clinical diagnostic criteria for PDD

Prior to the development of the MDS-proposed clinical diagnostic criteria (10), PD patients were diagnosed with dementia according to the DSM-IV criteria (42) on the basis of ‘dementia due to other general medical conditions’. Unfortunately, within these criteria, the section devoted to PDD is rather generic and imprecise, with reference to cognitive and motor slowing, executive dysfunction, impairment in memory retrieval and frequent exacerbation by depression. A comprehensive, systematic review of the literature related to the epidemiological, cognitive and neuropsychiatric motor and other clinical features, ancillary examinations, and clinico-pathological correlations enabled the MDS Task Force to propose clinical criteria for the diagnosis of possible and probable PDD (10).

**Figure 1:**
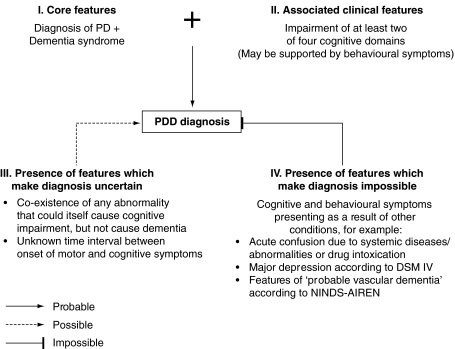
Parkinson's disease dementia (PDD) diagnosis overview based on the 2007 Movement Disorder Society guidelines (9,10)

The MDS Task Force proposed four clusters of features requiring sequential consideration to determine whether a diagnosis of PDD is probable, possible or impossible (Figure 1). Following the development of these criteria for PDD, the MDS Task Force subsequently published a recommended algorithm for diagnosing PDD (9). Thus, two versions of the MDS Task Force's recommendations exist: one tailored to the needs of clinicians requiring a simple, practical, screening tool in the office or at the bedside, which is summarised in Table 1 (9) and another, a more detailed approach for clinical monitoring, research studies or clinical trials (10). The shorter algorithm for clinicians comprises five criteria which, if all present, lead to a diagnosis of PDD.

**Table 1:** A simple algorithm for clinician diagnosis of PDD, as recommended by the MDS Task Force

	Criteria	Assessment
1	A diagnosis of PD	Queen's Square Brain Bank Criteria
2	PD developed prior to the onset of dementia	Patient/caregiver history or ancillary records
3	PD associated with a decreased global cognitive efficiency	MMSE < 26
4	Cognitive deficiency severe enough to impair daily life	Caregiver interview or pill questionnaire
5	Impairment of more than one cognitive domain	Impairment of at least two of the following domains
		Attention
		Executive function
		Visuo-constructive ability
		Memory

Table adapted from Dubois, et al. (9) with the permission of Wiley-Liss, Inc., a subsidiary of John Wiley & Sons, Inc. Presence of one of the following behavioural symptoms (apathy, personality changes, hallucinations, delusions or excessive daytime sleepiness) may support the diagnosis of probable PDD. Some behavioural symptoms can be assessed with the four-item Neuropsychiatric Inventory (hallucinations, depression, delusions and apathy). Refer to Figure 1 for concurrent features that may make PDD diagnosis uncertain/impossible. PDD, Parkinson's disease dementia; MDS, Movement Disorder Society; MMSE, Mini-Mental State Examination.

### Core features of probable PDD

The primary defining feature of PDD is dementia that develops in the setting of established PD (9,10). Therefore, the critical first step in the diagnosis process is to identify idiopathic PD, prior to the development of dementia. For a diagnosis of PDD, two core features must be present: (i) a diagnosis of PD according to the Queen Square Brain Bank criteria (43) and (ii) PD developed prior to the onset of dementia [PDD can be temporally distinguished from dementia with Lewy bodies (DLB) by the ‘1-year rule’; in PDD, motor symptoms develop at least 1 year before development of dementia, while in DLB, the motor symptoms occur no more than 1 year prior to the onset of dementia and frequently after the onset of dementia (44)].

In this case, a ‘dementia’ syndrome is defined as (i) impairment in at least two cognitive domains and (ii) cognitive deficiency severe enough to impair daily life (social, occupational or personal care) that must be independent of impairment because of PD motor symptoms. The MDS Task Force recommended that the Mini-Mental State Examination (MMSE) may be useful as a screening instrument for identifying cognitive impairment in PDD patients – the MMSE is a simple and universally applied scale that can be easily and quickly performed in the clinical setting (9). An MMSE score of 25 or below is proposed as the cut-off for identifying clinically significant cognitive impairment in this population (9).

### Associated clinical features of probable PDD

‘Associated clinical features’ are defined along four primary cognitive domains (attention, memory, executive and visuo-spatial functions) and a spectrum of behavioural disorders (9,10). The MDS Task Force recommended a number of tests from which the clinician could choose to assess the four primary cognitive domains (Table 2) and suggested that the four-item Neuropsychiatric Inventory, which covers hallucinations, depression, delusions and apathy, might be useful in assessing behavioural symptoms associated with PDD (9). A diagnosis of ‘probable’ PDD is made on the basis of a typical profile of cognitive deficits (i.e. impairment in at least two of the four cognitive domains supported by the presence of at least one behavioural symptom). If dementia exists in the presence of established PD, yet the associated clinical features are not considered ‘typical’ (e.g. the presence of a cognitive profile more consistent with AD), only ‘possible’ PDD should be diagnosed.

**Table 2:** Tests proposed by the MDS Task Force to assess cognitive deficits in the clinical setting (9)

Cognitive domain	Proposed tests	Cut-off scores
Attention	Serial 7s of the MMSE Repeatedly subtract 7 starting at 100	Two or more incorrect responses
	Months reversed Give months of the year backwards	Omission of two or more months
Executive function	Lexical fluency e.g. list words beginning with S in 1 min	Less than 9 words in a minute
	Clock-drawing test Draw clock with hands at ‘10 past 2’	Inability to draw clock or show time
Visuo-constructive ability	MMSE pentagons Copy two overlapping pentagons	Inability to draw pentagons
Memory	3-word recall of the MMSE Free recall of three words	Missing at least one word

Impairment of at least two of the four domains is required to support a diagnosis of probable Parkinson's disease dementia. MDS, Movement Disorder Society; MMSE, Mini-Mental State Examination.

### ‘Possible’ PDD

There are numerous other features that do not necessarily exclude PDD, but make the diagnosis of probable PDD uncertain (i.e. ‘possible’ PDD instead) (9,10). For example, if the time interval between the onset of motor and cognitive symptoms is unknown, it is difficult to distinguish whether a patient has DLB or PDD. History of medical or neurological comorbidities other than PD can also be associated with dementia (e.g. presence of significant cerebrovascular disease identified by imaging techniques) and their relevance must be considered when assigning a diagnosis.

Certain other conditions or diseases that can cause cognitive impairment and behavioural symptoms (e.g. infection, dehydration, vitamin deficiency or hormonal disturbances) make a reliable PDD diagnosis impossible and must be ruled out (9,10). Similarly, delirium and cognitive impairment secondary to PD treatments, the most common examples being anticholinergics, dopamine replacement therapies and benzodiazepines, must also be considered. A diagnosis of dementia can generally be made only in the absence of major depression, as the presence of significant depressive symptoms can impact on neuropsychological performance. Yet, given that depression is frequently concurrent in patients with PD (45), it should not be automatically considered a criterion for exclusion.

## Guidelines for management of PDD

As recognition of PDD as an independent dementia syndrome increases, potential therapies are becoming the focus of research efforts. Several guidelines/recommendations on the therapeutic management of PDD have been published since 2006 (Table 3) (38–41).

**Table 3:** Guidelines/recommendations published to date on the use of cholinesterase inhibitors for the symptomatic treatment of PDD

		Clinical evidence (class)		Recommendation (level)	
Authors	Task Force	Rivastigmine	Donepezil	Rivastigmine	Donepezil
Horstink et al. (39)	EFNS and MDS-ES	I	II	A	C
Waldemar et al. (40)	EFNS	I	–	A	–
Miyasaki et al. (38)	AAN	II	I and II	B	B
Maidment et al. (41)*	Cochrane	Yes	No	Yes	No

* One rivastigmine trial was the sole study identified that met the Cochrane inclusion criteria. The authors concluded that rivastigmine improves cognition and activities of daily living. *Clinical Evidence*: Class I–IV, strongest to weakest clinical evidence. *Recommendation*: Level A (established as effective, and should be used; based generally on at least two consistent class I studies) through to level U (data inadequate or conflicting, not recommended; based on studies not meeting criteria for class I–III). MDS-ES, European section of the MDS; PDD, Parkinson's disease dementia; EFNS, European Federation of Neurological Societies; AAN, American Academy of Neurology.

### EFNS Task Force recommendations

A joint task force of the EFNS and the European section of the MDS provided their recommendations for the therapeutic management of PD in 2006, including a section devoted to the management of non-motor problems in PD, such as dementia (39). Although they acknowledged that cognitive improvements in patients with PDD treated with cholinesterase inhibitors were modest, they classified clinical evidence with rivastigmine and donepezil as class I and II studies respectively. For overall management, they recommended both discontinuation of medications that might impair cognition (e.g. anticholinergics and amantadine) and the addition of cholinesterase inhibitor therapy either with rivastigmine (level A) or with donepezil (level C) (39). Additionally, the authors recommended that the addition of cholinesterase inhibitor therapy with rivastigmine (level B) or donepezil (level C) may also help in the treatment of psychosis in this population (39).

### Cochrane report

In a Cochrane meta-analysis on the use of cholinesterase inhibitors in PDD (41), the large, randomised, double-blind, placebo-controlled study of rivastigmine involving 541 patients was the sole study identified that met the inclusion criteria defined in the Cochrane Collaboration Handbook (46). The authors concluded that this clinical study provided clear evidence that rivastigmine has a beneficial effect on cognition and, to a lesser extent, ADLs in patients with PDD. In general, rivastigmine was well tolerated and no unexpected safety issues were reported. Adverse events were predominantly cholinergic in nature, the most frequent being nausea, vomiting, tremor and diarrhoea (which affected 29.0%, 16.6%, 10.2% and 7.2% of patients in the rivastigmine group *versus* 11.2%, 1.7%, 3.9% and 4.5% of those in the placebo group respectively). Adverse events were the primary reason for study discontinuation and resulted in the withdrawal of 17.1% of patients from the rivastigmine-treated group and 7.8% of patients in the placebo group. Tremor was usually dose-titration related, rarely severe (only one case of severe tremor was reported) and did not result in significant increases in concomitant dopaminergic medication, worsening of movement disorder assessments [Unified Parkinson's Disease Rating Scale (UPDRS) part III score] or study discontinuations (11).

### AAN practice parameter

In 2006, the dementia section of the American Academy of Neurology's (AAN) evidence-based practice parameters provided treatment recommendations for patients with PDD (38). The AAN guidelines concluded that the cholinesterase inhibitors, rivastigmine and donepezil, are probably effective in improving cognitive function and should be considered for the treatment of dementia in PD (level B). However, the AAN Subcommittee concluded that the magnitude of their benefit is modest [based on the number needed to treat to obtain clinically meaningful (moderate or marked) improvement on the Alzheimer's Disease Cooperative Study-Clinical Global Impressions of Change (ADCS-CGIC) with rivastigmine] and tremor may be exacerbated. These recommendations were based on three clinical studies summarised in Table 4.

**Table 4:** Cholinesterase inhibitor trials considered in the development of the AAN recommendations for the treatment of PDD

						Observed benefits		
	References	Indication	No. of patients	Study design	Study duration (weeks)	Cognition	ADL	Behaviour
Rivastigmine	Emre et al. (11)	PDD	541	Double-blind,placebo-controlled	24	+	+	+
Donepezil	Aarsland et al. (33)	PDD	14	Double-blind,placebo-controlled, crossover	10	+	ND	−
	Ravina et al. (47)	PDD	22	Double-blind,placebo-controlled, crossover	10	−*	ND	−

* Although a statistically significant benefit was observed on the study's secondary cognitive measure (Mini-Mental State Examination), there was no statistically significant benefit of donepezil treatment on the primary cognitive measure (ADAS-cog). PDD, Parkinson's disease dementia; AAN, American Academy of Neurology; ADL, activities of daily living; ADAS, Alzheimer's Disease Assessment Scale; ND, not determined.

+: Significant benefit observed in treated patients vs. placebo.

−: No significant benefit observed in treated patients vs. placebo.

## Conclusion

The introduction of guidelines for the diagnosis of dementia associated with PD represents an important milestone in its recognition as a distinct disease entity. It is imperative that PDD is recognised and accurately diagnosed by clinicians so that patients with this condition can benefit from appropriate treatment.

Currently, the cholinesterase inhibitor rivastigmine is approved for this condition. However, there remains a need to continue research into new and better treatments, in particular those that affect key disease mechanisms (e.g. α-synuclein aggregation) or prevent or delay patients with MCI-PD from progressing to PDD.
